# In the absence of UCP1-mediated diet-induced thermogenesis, obesity is augmented even in the obesity-resistant 129S mouse strain

**DOI:** 10.1152/ajpendo.00020.2019

**Published:** 2019-02-26

**Authors:** Ineke H. N. Luijten, Helena M. Feldmann, Gabriella von Essen, Barbara Cannon, Jan Nedergaard

**Affiliations:** Department of Molecular Biosciences, The Wenner-Gren Institute, Stockholm University, Stockholm, Sweden

**Keywords:** 129S, brown adipose tissue, diet-induced thermogenesis, obesity, UCP1

## Abstract

The attractive tenet that recruitment and activation of brown adipose tissue (BAT) and uncoupling protein 1 (UCP1) could counteract the development of obesity and its comorbidities in humans has been experimentally corroborated mainly by experiments demonstrating that UCP1-ablated mice on a C57Bl/6 background (exempt from thermal stress) become more obese when fed a high-fat diet. However, concerns may be raised that this outcome of UCP1 ablation is restricted to this very special inbred and particularly obesity-prone mouse strain. Therefore, we have examined to which degree UCP1 ablation has similar metabolic effects in a mouse strain known to be obesity resistant: the 129S strain. For this, male 129S2/sv or 129SV/Pas mice and corresponding UCP1-knockout mice were fed chow or a high-fat or a cafeteria diet for 4 wk. The absence of UCP1 augmented obesity (weight gain, body fat mass, %body fat, fat depot size) in high-fat diet- and cafeteria-fed mice, with a similar or lower food intake, indicating that, when present, UCP1 indeed decreases metabolic efficiency. The increased obesity was due to a decrease in energy expenditure. The consumption of a high-fat or cafeteria diet increased total BAT UCP1 protein levels in wild-type mice, and correspondingly, high-fat diet and cafeteria diet-fed mice demonstrated increased norepinephrine-induced oxygen consumption. There was a positive correlation between body fat and total BAT UCP1 protein content. No evidence for diet-induced adrenergic thermogenesis was found in UCP1-ablated mice. Thus, the obesity-reducing effect of UCP1 is not restricted to a particular, and perhaps not representative, mouse strain.

## INTRODUCTION

The tenet that recruitment and activation of brown adipose tissue (BAT) and uncoupling protein 1 (UCP1), the thermogenic protein located in BAT mitochondria, could provide a mechanism to decrease metabolic efficiency and thus counteract weight gain is attractive. Although this tenet had already been implied in 1979 by Rothwell and Stock (21), direct experimental evidence for a significant role for UCP1 in counteracting obesity has been limited to the metabolic consequences of ablation of UCP1. Indeed, UCP1 ablation does augment diet-induced obesity, at least when mice are housed at thermoneutrality (8). However, although this obesity-promoting effect of UCP1 ablation has been reported from different laboratories (7, 8, 14, 22, 28), a serious limitation of the existing studies has been that the study object has been restricted to a single mouse strain: the notoriously obesity-prone C57Bl/6 mouse. Thus, the effect of UCP1 ablation on obesity may be limited to this very special strain.

Metabolically, mouse strains are very diverse, and the C57Bl/6 and AKR/J strains are prone to the development of obesity, whereas the 129Sv and SWR/J and A/J strains are obesity-resistant resistant (5, 27). These differences have been discussed to be due to genetic variations in macronutrient preference, adipocyte sensitivity to insulin, and metabolic efficiency, etc., but the differences may include issues related to BAT UCP1 (4, 5, 24, 27). Clearly, it is thus of importance to examine to which degree the obesity-inducing effect of UCP1 ablation is a phenomenon also observable in strains that do not readily become obese. A corresponding question is whether UCP1 is the only mediator of diet-induced thermogenesis or whether other mechanisms are present in other less obesity-prone strains. Evidently, such data are important to allow for possible translation of UCP1 effects to human conditions, as metabolic effects of UCP1 restricted to a single obesity-prone mouse strain would hardly be transferable to humans.

Therefore, we examine here the contribution of BAT to facultative diet-induced thermogenesis in two obesity-resistant 129s mouse strains, i.e., strains in the opposite extreme of metabolic regulation from the C57Bl/6 mice. We show that, similarly to what is the case in C57Bl/6 mice, diet-induced thermogenesis is mediated solely by BAT UCP1 in the 129s mice and that even in these mice obesity is augmented in the absence of UCP1.

## METHODS

### 

#### Animals and diets.

Male 10- to 14-wk-old 129S2/sv wild-type and UCP1-knockout (KO) mice on a 129S2/sv background [bred from the mice generated by Enerbäck et al. (6) and backcrossed to 129S2/sv for at least 10–11 generations] were single-caged and housed at 30°C with a 12:12-h dark-light cycle (lights on at 8 AM) and free access to water and chow diet (R70 Labfor; Lantmännen). After a 2-wk acclimation period (*day 1*), body composition was measured by MRI (see below). The next day (*day 2*), mice were assigned to continue either on the chow diet or switch to a high-fat diet (HFD; D12451; Research Diets). An overview of the macronutrient composition of the diets is given in Table 1. The animals were maintained on the chow or HFD for 29 days. Body weight and gross food intake were measured twice every week. The cage was examined for food spillage, and the measurements were corrected for this.

**Table 1.** Energy content and macronutrient composition per 100 g of the experimental diets

	Energy, kJ	Protein, g	Carbohydrate, g	Fat, g
Chow diet	1,255	14.5	60	4.5
HFD	1,980	24	24	41
Cafeteria diet biscuit/paste	1,900/1,841	6.5/10.2	60/62	21/18

HFD, high-fat diet.

In a separate experiment, male 6- to 9-wk-old 129SV/pas wild-type and UCP1^−/−^ mice on a 129SV/pas background (also backcrossed for ≥10 generations) were single-caged and housed at 30°C with a 12:12-h dark-light cycle and free access to water. The animals were fed either the chow diet, HFD (as above), or a simplified cafeteria diet consisting of digestive biscuits (LU digestive) and almond paste (Odense mandelmassa) in addition to chow ad libitum for 35 days (see Table 1 for diet composition). Body weight and food intake were measured three times/wk. For the cafeteria diet, the intake of each component was measured separately, and the total food energy was calculated from this.

For all experimental 129SV/pas groups, gross food intake was determined in grams per day and converted to kilojoules per day based on the energy density of the diet (Table 1). During the 35-day experimental period, the measurements outlined below were executed. In experimental *week 5*, the animals were euthanized by CO_2_ and cervical dislocation, and organs were collected.

#### Body composition and metabolic efficiency.

Body composition [fat mass (g) and lean mass (g)] of each 129S2/Sv mouse was measured by MRI (EchoMRI100; Echo Medical Systems, Houston, TX) on experimental *days 1*, *9*, and *26*. The metabolic efficiency [fat gained (kJ)/energy consumed (kJ)] of the 129S2/sv mice was calculated as the change in body composition from experimental *days 1–9* or *9–26* [change in fat mass (in kJ, 39 kJ/g) + change in lean mass (in kJ, 5 kJ/g)] divided by gross food intake (in kJ) from experimental *days 1–9* or *9–26* multiplied by 100 (3). Body composition of 129SV/Pas mice was measured once in experimental *week 5*.

#### Indirect calorimetry.

O_2_ consumption and CO_2_ production of the animals were measured every 2 min under a constant air flow (1,000 ml/min) in temperature-controlled metabolic chambers (INCA System; Somedic, Hörby, Sweden). The animals were placed in the metabolic chambers in their home cages and were kept at 30°C on a 12:12-h dark-light cycle with free access to food (chow or HFD) and water. Measurements in the metabolic chambers were made on experimental *days 1–4*, *9–11*, and *26–28* for the 129S2/sv mice. During the first time period (*days 1–4*), all mice were removed from the metabolic chambers on *day 2* for <5 min to note body weight and food intake and to provide the HFD group with the HFD. For each time period, the first 5 h of measurements were excluded from calculations.

Respiratory quotient (RQ) was calculated by dividing CO_2_ produced by O_2_ consumed per time point. The theoretical RQ of the HFD was calculated by converting the relative macronutrient composition of the diet to kcal (20% protein, 35.1% carbohydrates, and 44.9% fat) and using these values to calculate the contribution of the RQ of each macronutrient (0.81 for protein, 1 for carbohydrates, and 0. 71 for fat), yielding an RQ of 0.83.

Energy expenditure in joule (J) per minute was calculated for each time point according to the following equation: 16.3 × O_2_ consumption (ml/min) + 4.57 × RQ × O_2_ consumption (ml/min) (2). Values for energy expenditure were converted to watts (W). Average energy expenditure was calculated from *dark period 2* to *dark period 4* for experimental *days 1–4* (due to changing of the diet in *light period 1*) and from *dark period 1* to *dark period 3* for experimental *days 9–11* and *26–28*. Δenergy expenditure was calculated per diet group for experimental *days 26–28* by subtracting the average energy expenditure of the UCP1-KO mice from the average energy expenditure of the WT mice per measurement.

In experimental *week 5*, resting metabolic rate (RMR) was determined in the 129SV/pas mice. Conscious animals were placed in the metabolic chambers at 30°C in the absence of food for 3 h. The first hour of measurements was excluded from calculations. The RMR was calculated by averaging the three lowest points in O_2_ consumption measurements.

It may be noted that although the mice remained in their home cages and had unchanged food, light, and temperature conditions during the calorimetric measurements, they still responded to the altered environment, as can be seen, e.g., from the change in eating behavior during these time periods.

#### Adrenergically induced oxygen consumption.

In experimental *week 5*, 129SV/pas mice were sedated with 90 mg/kg ip pentobarbital (APL), and basal O_2_ consumption was measured for 30 min at 33°C in the temperature-controlled metabolic chambers. Subsequently, the mice were taken out of the chambers, and 1 mg/kg norepinephrine bitartrate salt monohydrate (NE; Sigma) was injected subcuteanously. O_2_ consumption was then measured for another 60–80 min. The average of the basal O_2_ consumption was set to zero, and the O_2_ consumption trace was expressed as O_2_ consumption minus average basal O_2_ consumption. UCP1-dependent O_2_ consumption was calculated by subtracting the average O_2_ consumption trace of the UCP1-KO mice from the average O_2_ consumption trace of the WT mice per diet group.

#### Protein analyses.

Total interscapular BAT (IBAT) and total inguinal WAT (ingWAT) were collected, weighed, and snap-frozen in liquid nitrogen. Samples were stored at −80°C until further analysis. IBAT and ingWAT tissues were homogenized in RIPA buffer (50 mM Tris·HCl, pH 7.4, 1% Triton X-100, 150 mM NaCl, and 1 mM EDTA) containing protease (cOmplete Mini; Roche) and phosphatase (5 mM Na fluoride, 1 mM Na orthovanadate) inhibitors. Total protein concentration was determined with the Lowry method and spectrophotometry at λ = 750 nm (Beckman DU 530). Samples were diluted in sample buffer [50 mM Tris·HCl, pH 6.8, 2% (wt/vol) SDS, 50 mM dl-dithiothreitol (DTT), 10% glycerol, and 0.02% bromphenol blue] and boiled at 95°C for 5 min. Proteins were separated by SDS-PAGE on an 11–12% polyacrylamide gel and transferred to a PVDF membrane (Bio-Rad) using a semidry electrophoretic transfer cell (Bio-Rad Trans-Blot SD; Bio-Rad Laboratories). Subsequently, the membranes were blocked in 5% (wt/vol) dried milk (Semper) in TBST (Tris-buffered saline with Tween 20) for 1 h at room temperature, after which they were probed with a primary anti-UCP1 antibody [rabbit polyclonal, produced in laboratory, 1:15,000 (for protein isolated from IBAT) or 1:3,000 (for protein isolated from ingWAT) in 5% milk in TBST)] overnight at 4°C. They were then incubated with a secondary goat anti-rabbit HRP-conjugated antibody [Cell Signaling Technology 7074, 1:5,000 (for protein isolated from IBAT) or 1:3,000 (for protein isolated from ingWAT) in 5% milk in TBS-T] for 1 h at room temperature. UCP1 was visualized by chemiluminescence using Western blotting detection reagents (ECL kit; GE Healthcare Life Sciences) and imaged with a charge-coupled device camera. Multi Gauge version 3.0 Software (Fujifilm) was used for quantification. Samples loaded on different membranes were compared by normalization of band intensity with a standard sample loaded on all membranes.

## RESULTS

### 

#### Even in 129S2/sv mice, UCP1 ablation leads to increased obesity and higher metabolic efficiency.

**Fig. 1.**
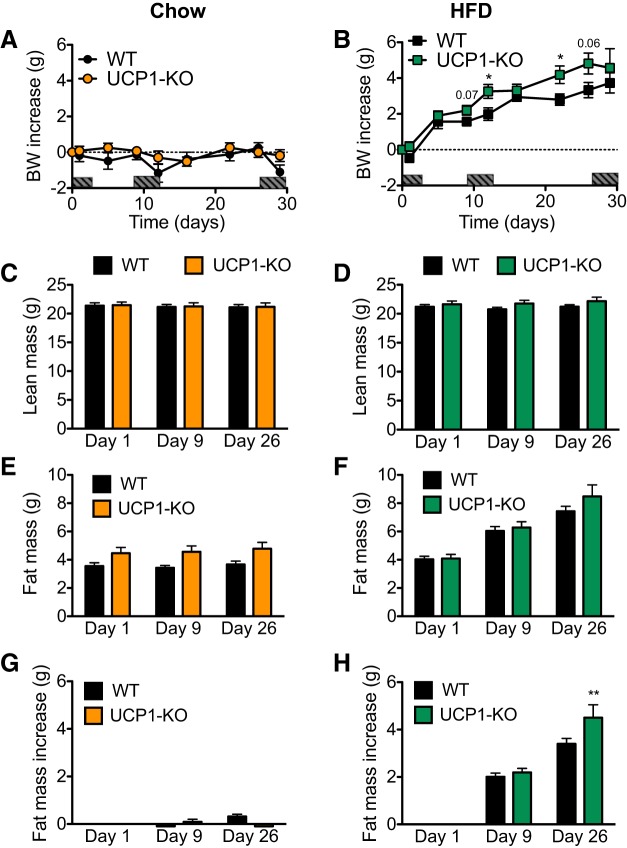
The absence of uncoupling protein 1 (UCP1) increases body weight gain and fat accumulation in mice fed a high-fat diet (HFD). Data from 129S2/sv mice kept at thermoneutrality. *A* and *B*: body weight (BW) increase in wild-type (WT) and UCP1-knockout (KO) mice fed chow (*A*) or HFD (*B*) for 30 days. Dark rectangles on the *x*-axes indicate time periods when the animals were housed in the metabolic chambers. *C* and *D*: total lean mass of the mice in *A* and *B* on experimental *days 1*, *9*, and *26*. *E* and *F*: total fat mass of the mice in *A* and *B* on experimental *days 1*, *9*, and *26*. *G* and *H*: fat mass increase per mouse. Data are represented as means ± SE; *n* = 7. *P < 0.05 and **P < 0.01; 2-way ANOVA with Bonferroni posttest, significance of genotype not significant (*A* and *B*); Student’s *t*-test, significance within each time point between genotypes (*A* and *B*); 2-way ANOVA with Bonferroni posttest, significance within each time point between genotypes (*C*–*H*).

To determine the metabolic significance of UCP1 in the obesity-resistant 129S2/sv mice, we examined the effects of UCP1 ablation on body composition and energy metabolism. We housed wild-type (WT) mice and UCP1-KO mice at 30°C and provided them with either a chow or a high-fat diet (HFD) for 1 mo. There were no differences in age or body weight between the four groups at the start of the experiment (Table 2). The mice that were fed a chow diet did not gain weight in either the presence or absence of UCP1 and maintained a stable lean mass and fat mass throughout the experimental period (Fig. 1, *A*, *C*, *E*, and *G*). In contrast, as shown earlier, the obesity-prone C57Bl/6 mice gain both body weight and fat mass even with a chow diet under these conditions (8). WT mice fed the HFD gained ∼3.5 g during the 30-day period (Fig. 1*B*), i.e., only about half of the amount that C57Bl/6 mice would gain under similar conditions (≈6 g) (8). Thus, the 129S2/sv WT mice were relatively obesity resistant under thermoneutral conditions.

**Table 2.** Initial body weight (g) and age (wk) for 129S2/sv mice

	Chow		HFD	
129S2/sv	Age	Weight	Age	Weight
WT	13.1 ± 0.4	24.9 ± 0.8	13.8 ± 0.7	25.2 ± 0.5
UCP1-KO	14.1 ± 0.4	26.1 ± 1.0	13.8 ± 0.7	26.0 ± 0.9

Data are represented as means ± SE. HFD, high-fat diet; UCP1-KO, uncoupling protein 1-knockout; WT, wild type. No statistically significant differences.

In the HFD-fed mice, the absence of UCP1 led to a slight but consistently larger increase in weight gain (Fig. 1*B*). Because there were no differences between the lean mass of HFD-fed WT and UCP1-KO mice, the increase in weight gain in HFD-fed UCP1-KO mice compared with HFD-fed WT mice could be attributed solely to a significantly higher increase in fat mass in the UCP1-KO mice (Fig. 1, *D*, *F*, and *H*). Thus, even in the obesity-resistant 129S2/sv mice, the absence of UCP1 promoted additional obesity.

**Fig. 2.**
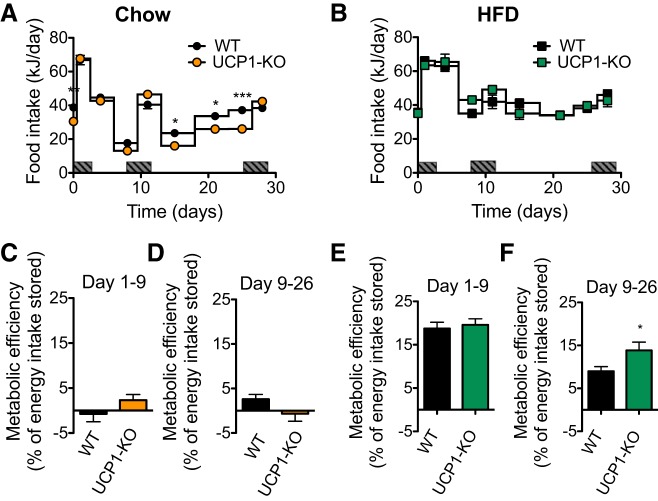
Metabolic efficiency is increased in the absence of uncoupling protein 1 (UCP1). Data from the 129S2/sv mice. *A* and *B*: gross energy intake in kJ/day of wild-type (WT) and UCP1-knockout (KO) mice fed a chow (*A*) or high-fat diet (HFD; *B*) for 30 days. Dark rectangles on the x-axes indicate time periods when the animals were housed in the metabolic chambers. *C* and *E*: metabolic efficiency calculated as the %ingested energy that is stored in the body in WT and UCP1-KO mice fed a chow diet (*C*) or HFD (*E*) for experimental *days 1–9*. *D* and *F*: metabolic efficiency as in *C* and *E* for experimental *days 9–26*. Data are represented as means ± SE; *n* = 7. *P < 0.05 and ***P < 0.001, 2-way ANOVA with Bonferroni posttest, significance between genotypes shown for each time point (*A* and *B*); Student’s *t*-test (*C*–*F*).

Although we found no differences in body weight and body composition between chow-fed WT and UCP1-KO mice, the WT mice had a slightly higher food intake in the last half of the experimental period (Fig. 2*A*). However, standard calculations of metabolic efficiency (the relation between the increase in body energy content and amount of food energy taken in) revealed no differences between the genotypes (Fig. 2, *C* and *D*). The calculated values for metabolic efficiency were close to zero, which indicated that chow-fed mice of both genotypes did not store the energy they consumed but rather fully expended it. However, it is noteworthy that the UCP1-KO mice were able to maintain their body energy content during this period on an energy intake that was 16% lower than that of the WT mice (*P* < 0.001). This in itself indicates that the presence of UCP1 is associated with a higher ongoing metabolism and in this respect with a decreased metabolic efficiency, although the formal calculations of metabolic efficiency cannot reveal this, as zero divided by any number will remain zero.

The increase in weight gain we observed in UCP1-KO mice fed HFD compared with the WT mice was not due to differences in food intake between the two groups (Fig. 2*B*). At the start of the experimental period, HFD-fed mice of both genotypes stored a considerable amount of their energy intake as fat, ∼19% (Fig. 2*E*). Between *days 9* and *26* of HFD consumption, a significant difference in metabolic efficiency between the WT and UCP1-KO mice became visible (Fig. 2*F*). Thus, during prolonged HFD feeding, the UCP1-KO mice became more energetically efficient than the WT mice and thus stored more of what they ate.

#### Shifted circadian rhythmicity of substrate utilization in the absence of UCP1.

**Fig. 3.**
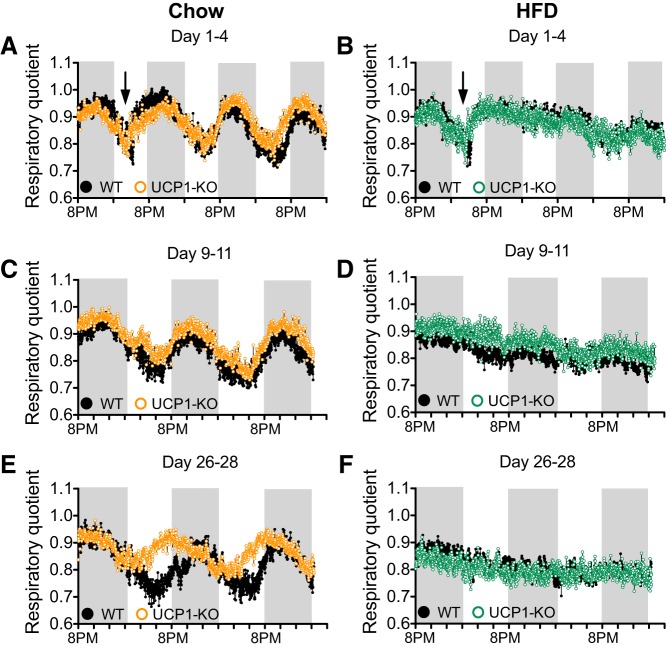
High-fat diet (HFD) feeding, but not the presence of uncoupling protein 1 (UCP1), affects substrate utilization. Data from the 129S2/sv mice. *A* and *B*: respiratory quotient calculated as V̇co_2_/V̇o_2_ of wild-type (WT) and UCP1-knockout (KO) mice fed chow (*A*) or HFD (*B*) for experimental *days 1–4*. Black arrows indicate opening of the metabolic chambers and providing the mice in *B* with the HFD. Gray background indicates dark phase. *C* and *D*: respiratory quotient as in *A* and *B* for experimental *days 9–11*. *E* and *F*: respiratory quotient as in *A* and *B* for experimental *days 26–28*. Data are represented as means (SE not shown); *n* = 7.

We determined the effects of the absence of UCP1 on the respiratory quotient (RQ) (as an indicator for substrate utilization). For this purpose, the mice were placed in temperature-controlled metabolic chambers kept at 30°C during *days 1–4*, *9–11*, and *26–28* of the experimental period. Measurements were made of CO_2_ production and O_2_ consumption, and the ratio was calculated (i.e., RQ). As expected, mice of both genotypes fed chow initially showed a clear circadian rhythm of substrate utilization, which was visible as RQ values close to 1 during the dark period, indicating the utilization of carbohydrates as substrate, and RQ values close to 0.7 during the light period, indicating the utilization of fat as substrate (Fig. 3*A*). The WT chow-fed mice continued to show this pattern of circadian substrate utilization (Fig. 3, *C* and *E*). Surprisingly, measurements made during *days 26–28* showed a marked shift in circadian rhythm in the UCP1-KO mice (Fig. 3*E*). The shift from lipid toward carbohydrate utilization occurred much earlier during the light phase; i.e., the UCP1-KO mice started eating earlier. Such a shift in daily metabolic rhythm has not been observed previously in UCP1-KO mice; it is remarkable that it develops spontaneously while the mice become slightly older (from 13 to 17 wk old). It does have its parallel in the behavior of mice with denervated IBAT. In these mice, the eating period is correspondingly advanced to earlier in the light phase (9).

During the first light period the metabolic chambers were opened (black arrows in Fig. 3, *A* and *B*), and for half of the mice the chow diet was replaced with the HFD. HFD feeding affected substrate utilization immediately, as the mice receiving the HFD did not show a circadian rhythm of substrate utilization any more but rather maintained high RQ values (Fig. 3*B*). The maintained RQ value approaches the calculated RQ for the HFD (≈0.83; see methods), implying that during all times of the day, the energy is obtained directly from the food. Indeed, the provision of a HFD leads to food intake even during the light phase (9, 13, 18). The absence of a circadian rhythm in RQ was maintained in the HFD-fed mice throughout the treatment period, with no effect of genotype (however, measurements of energy expenditure also showed that HFD-fed UCP1-KO mice shifted their circadian rhythm during *days 26–28*; see below).

#### Lower energy expenditure in the absence of UCP1.

**Fig. 4.**
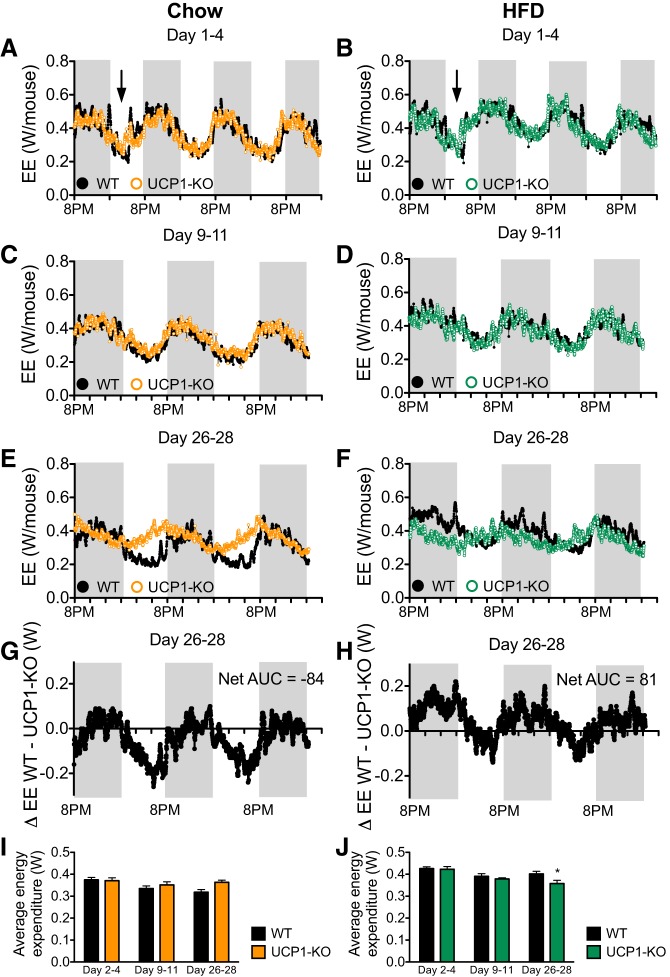
The absence of uncoupling protein 1 (UCP1) shifts circadian rhythm and reduces energy expenditure upon high-fat diet (HFD) feeding. Data from the 129S2/sv mice. *A* and *B*: energy expenditure for experimental *days 1–4* calculated in watt/total animal of wild-type (WT) and UCP1-knockout (KO) mice fed a chow (*A*) or HFD (*B*). Black arrows indicate opening of the metabolic chambers and providing the mice in *B* with the HFD. Gray background indicates dark phase. *C* and *D*: energy expenditure as in *A* and *B* for experimental *days 9–11*. *E* and *F*: energy expenditure as in *A* and *B* for experimental *days 26–28*. *G* and *H*: Δaverage energy expenditure between WT and UCP1-KO mice as in *E* and *F*. The calculated net area under the curves (AUC) are indicated. *I* and *J*: average energy expenditure calculated from *A*–*F* of WT and UCP1-KO mice fed a chow (*G*) or HFD (*H*). Data in *A*–*F* are represented as means (SE not shown); data in *I* and *J* are represented as means ± SE; *n* = 7. *P < 0.05; 2-way ANOVA with Bonferroni posttest, significance between genotypes shown for each time period (*I* and *J*).

In parallel to determining RQ values, we calculated energy expenditure from the O_2_ and CO_2_ data. During *days 1–4* and *9–11*, animals in all groups showed the expected circadian energy expenditure pattern, consisting of low energy expenditure in the light phase and an increased energy expenditure in the dark phase (Fig. 4, *A*–*D*). Analysis of the average energy expenditure for these time periods revealed that HFD-fed mice of either genotype in general expended more energy than chow-fed mice of either genotype [*days 2–4*: WT chow vs. WT HFD (*P* < 0.01), UCP1-KO chow vs. UCP1-KO HFD (*P* < 0.05); *days 9–11*: WT chow vs. WT HFD (*P* < 0.01), UCP1-KO chow vs. UCP1-KO HFD, not significant; Fig. 4, *G* and *H*]. No differences in energy expenditure were found between genotypes in these the two early measuring periods (Fig. 4, *A*–*D*).

However, during the last studied period (*days 26–28*), energy expenditure in HFD-fed mice was significantly lower in mice without UCP1 (Fig. 4, *F*, *H*, and *J*). This was not secondary to a lowered food intake (Fig. 2*B*). From the total difference between the areas under the curves in the “Δ” graphs (WT − UCP1-KO; Fig. 4, *G* and *H*), it becomes clear that HFD feeding leads to a consistently higher energy expenditure in both the light and the dark phases. Because HFD-fed mice also eat during the light phase, this thus indicates that it is the meal-induced facultative thermogenesis that is reduced (or even abolished) due to the absence of UCP1.

UCP1-KO mice fed a HFD thus fail to develop diet-induced thermogenesis of the magnitude seen in WT mice, and this with time leads to an increase in fat mass.

#### HFD feeding increases total IBAT UCP1 protein amount.

**Fig. 5.**
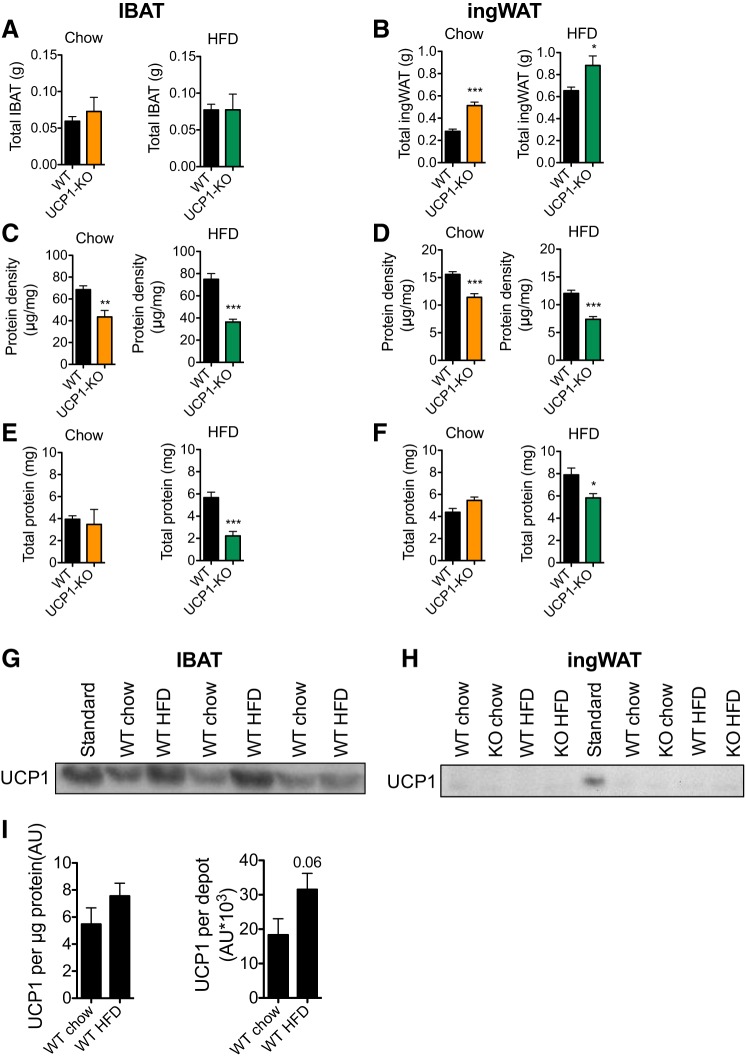
Consumption of a high-fat duet (HFD) increases total interscapular brown adipose tissue (IBAT) uncoupling protein (UCP1) protein levels. Data from the 129S2/sv mice, examined on experimental *day 29*. *A* and *B*: total IBAT (*A*) and inguinal white adipose tissue (ingWAT; *B*) tissue weights of wild-type (WT) and UCP1-knockout (KO) mice fed a chow or HFD (*n* = 7). *C* and *D*: protein density of IBAT (*C*) and ingWAT (*D*) tissues as in *A* and *B*. *E* and *F*: total protein in IBAT (*E*) and ingWAT (*F*) tissues as in *A* and *B*. *G* and *H*: representative immunoblot showing UCP1 protein in IBAT (*G*) and ingWAT (*H*) homogenates. Standard = mixture of BAT homogenates. *I*: quantification of data as in *G* showing UCP1/μg protein and calculated total UCP1/depot (*n* = 4). Data are represented as means ± SE. *P < 0.05, **P < 0.01, ***P < 0.001; Student’s *t*-test.

It is implicit from the above results that it is the absence of the recruitment of UCP1-dependent diet-induced thermogenesis in the UCP1-KO mice that is the cause of the development of obesity. Therefore, at the end of the experiment, on treatment *day 29*, we examined the mice for signs of diet-induced recruitment in UCP1-expressing tissues. We isolated the interscapular brown adipose tissue (IBAT) and the inguinal white adipose tissue (ingWAT) depots. Although we observed no differences in total IBAT weight between the groups (Fig. 5*A*), total ingWAT weight was significantly higher in HFD-fed mice compared with chow-fed mice [WT chow vs. WT HFD (*P* < 0.001), UCP1-KO chow vs. UCP1-KO HFD (*P* < 0.01); Fig. 5*B*]. The weight of the ingWAT was higher in UCP1-KO mice compared with WT mice not only in the HFD-fed mice but also in the chow-fed mice (Fig. 5*B*). That this higher wet weight was due mainly to increased lipid content can be deduced from the fact that protein density was decreased in the ingWAT of UCP1-KO mice fed either diet (Fig. 5, *C* and *D*). Thus, even the chow-fed UCP1-KO mice showed signs of increased adiposity.

As an indirect measure of tissue recruitment, we measured the total protein content in the adipose tissues. The content had increased due to HFD feeding in the WT mice [IBAT WT chow vs. WT HFD (*P* < 0.01), ingWAT WT chow vs. WT HFD (*P* < 0.001)], but no increase was seen in the UCP1-KO mice (Fig. 5, *E* and *F*).

As the most appropriate biochemical measure of thermogenic tissue recruitment, we examined UCP1 levels. We determined UCP1 protein per microgram of IBAT protein by immunoblotting and found a slight but nonsignificant increase in HFD-fed mice compared with chow-fed mice (Fig. 5, *G* and *I*). However, the total amount of UCP1 protein better reflects the total heat-producing/energy-consuming capacity of the tissue. The total amount of UCP1 is calculated as the UCP1 protein per microgram of IBAT protein multiplied by the total amount of protein present in the IBAT (Fig. 5*E*). The total amount of UCP1 in the tissue was apparently nearly doubled in the HFD-fed mice vs. the chow-fed mice (but not statistically significantly increased, *P* = 0.06; Fig. 5*I*). Thus, in the 129S2/sv mouse strain, similarly to the C57Bl/6 mouse strain (7, 8), a HFD regime leads to increased thermogenic capacity in the BAT, as estimated from total UCP1 protein levels.

In ingWAT, no UCP1 protein was detectable in the WT mice (Fig. 5*H*), and therefore, this tissue could not contribute to UCP1-dependent, diet-induced thermogenesis.

#### In 129SV/pas mice, both cafeteria- and HFD-fed UCP1-KO mice accumulate extra fat mass.

As shown above, in 129S2/sv mice fed a HFD, the absence of UCP1 increases metabolic efficiency and decreases energy expenditure and thereby leads to an increase in weight gain due to fat mass accumulation. To exclude that the importance of UCP1 for body weight regulation in obesity-resistant mice is restricted to the 129S2/sv substrain, we performed a similar experiment on 129SV/pas mice. These mice were also housed at 30°C for 1 mo. The 129SV/pas mice were provided with either a chow diet, a simplified cafeteria diet, or a HFD. The simplified cafeteria diet consisted of digestive biscuits and almond paste, between which the mice could freely chose in addition to chow; it may thus be considered a less artificial diet than the fully defined HFD. The cafeteria diet has a higher carbohydrate and lower fat content than the HFD but a comparable energy density (Table 1).

**Fig. 6.**
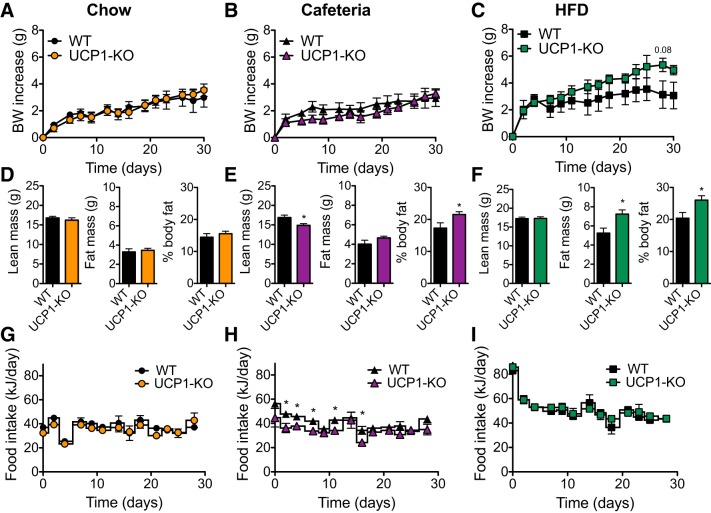
The absence of uncoupling protein (UCP1) affects body fat accumulation in high-fat diet (HFD)- and cafeteria-fed mice. Data from 129SV/pas mice housed at thermoneutrality for the duration of the study. *A*–*C*: body weight (BW) increase in wild-type (WT) and UCP1-knockout (KO) mice fed a chow (*A*), cafeteria (*B*), or HFD (*C*) for 30 days. *D*–*F*: body composition of the mice in *A*–*C* in experimental *week 5*. *G*–*I*: gross energy intake in kJ/day of the mice in *A*–*C*. Data are represented as means ± SE; *n* = 6. *P < 0.05, Student’s *t*-test.

At the start of the study, there was no difference in the age or weight between the chow-fed and the cafeteria-fed mice, but for technical reasons, the HFD-fed mice were on average 2 wk older than the chow-fed and cafeteria-fed mice, and therefore, they also weighed slightly more (Table 3). We found no differences in body weight increase, lean mass, fat mass, or food intake between the WT and UCP1-KO mice receiving a chow diet (Fig. 6, *A*, *D*, and *G*).

**Table 3.** Initial body weight (g) and age (weeks) for 129SV/pas mice

	Chow		Cafeteria		HFD	
129SV/pas	Age	Weight	Age	Weight	Age	Weight
WT	6.0 ± 0.0^a^	19.9 ± 1.0^a^^b^	6.0 ± 0.0^a^	20.6 ± 0.7^a^^b^	8.0 ± 0.0^b^	22.0 ± 0.4^a^^b^	
UCP1-KO	6.3 ± 0.2^a^	18.8 ± 0.8^a^	6.5 ± 0.2^a^	18.8 ± 0.9^a^	8.3 ± 0.2^b^	22.6 ± 0.8^b^	

Data are represented as means ± SE. HFD, high-fat diet; UCP1-KO, uncoupling protein 1-knockout; WT, wild type.

^a,b^ Superscripted letters are significantly different, *P* < 0.05, 1-way ANOVA with Tukey posttest.

There was no difference in body weight gain between genotypes in mice that received the cafeteria diet (Fig. 6*B*). However, the fat mass in cafeteria-fed UCP1-KO mice was significantly increased compared with the fat mass in chow-fed UCP1-KO mice (*P* < 0.01), and the absence of UCP1 led to a significant increase in the percentage of body fat at the end of the cafeteria diet treatment period (Fig. 6*E*). That this increase was not higher may be due to the lower food intake in the cafeteria-fed UCP1-KO mice during the first half of the experimental period (Fig. 6*H*). Indeed, the WT mice consumed an average of 200 kJ more than the UCP1-KO mice during the treatment period, whereas they showed a 25 kJ lower fat mass at the end of the treatment period (Fig. 6, *E* and *H*). This indicates a lower metabolic efficiency in the WT mice and thus that the absence of UCP1 increases metabolic efficiency in cafeteria-fed animals.

Similarly to what was the case in the 129S2/sv cohort, feeding the 129SV/pas mice a HFD had only marginal effects on body weight gain (Fig. 6*C*). Furthermore, the fat mass of HFD-fed UCP1-KO mice was significantly increased both as compared with the corresponding WT mice and even compared with cafeteria-fed UCP1-KO mice (*P* < 0.001) (Fig. 6, *E* and *F*). Thus, the presence of UCP1 prevented both cafeteria-fed and HFD-fed 129SV/pas mice from becoming as obese as they would be in the absence of UCP1.

#### Cafeteria and HFD feeding augments UCP1-dependent norepinephrine-induced thermogenesis without affecting UCP1-independent thermogenesis.

To examine whether the protection against diet-induced obesity observed above in WT versus UCP1-KO mice would be associated with a recruited UCP1-dependent thermogenic capacity, we determined adrenergically-induced O_2_ consumption. For this we examined the thermogenic response to a norepinephrine (NE) injection.

**Fig. 7.**
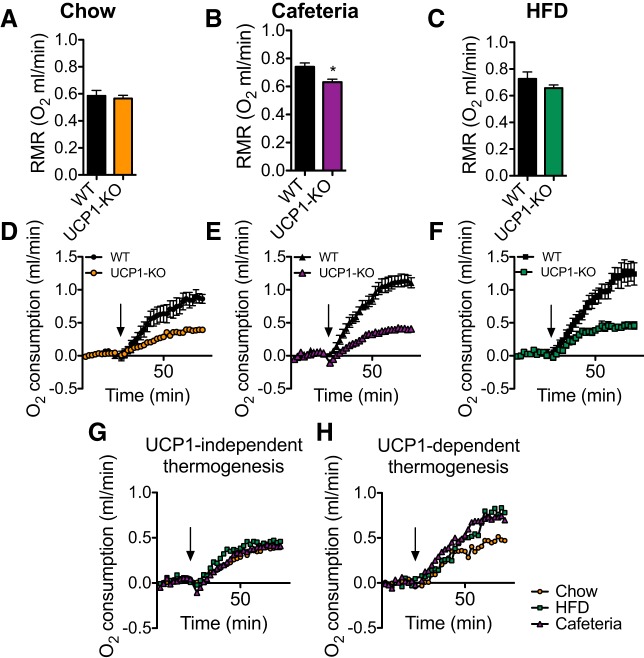
Uncoupling protein (UCP1)-dependent thermogenesis in cafeteria- and high-fat diet (HFD)-fed mice. Data from the 129SV/pas mice examined on experimental *day 31*. *A*–*C*: resting metabolic rate (RMR) of anesthetized wild-type (WT) and UCP1-knockout (KO) mice fed a chow (*A*), cafeteria (*B*), or HFD (*C*). *D*–*F*: norepinephrine (NE)-induced O_2_ consumption of mice in *A*–*C*, increase over basal O_2_ consumption. Black arrows indicate time of norepinephrine (NE) injection. *G*: UCP1-independent NE-induced thermogenesis calculated as in *D*–*F*. *H*: UCP1-dependent NE-induced thermogenesis calculated from *D*–*F*. Data are represented as means ± SE; *n* = 6. *P < 0.05; Student’s *t*-test.

Because the NE injection has to be performed in anesthetized mice, the metabolic rate of mice under anesthesia is recorded first. We found no differences in this basal O_2_ consumption rate between genotypes in chow-fed mice (Fig. 7*A*). However, in WT mice, the consumption of a cafeteria diet or a HFD significantly increased basal O_2_ consumption [WT chow vs. WT cafeteria (*P* < 0.01), WT chow vs. WT HFD (*P* < 0.05); Fig. 7, *A*–*C*]. UCP1-KO mice fed a cafeteria diet had a lower basal O_2_ consumption than WT mice (Fig. 7*B*). This may contribute to the fact that these mice had an increased metabolic efficiency compared with cafeteria-fed WT mice, as discussed above. Neither the cafeteria diet nor the HFD affected basal O_2_ consumption in the UCP1-KO mice (Fig. 7, *A*–*C*).

To measure the full capacity for UCP1-dependent thermogenesis, we then injected the anesthetized mice with NE. WT mice of all diet groups had a higher O_2_ consumption than the UCP1-KO mice, thus visualizing the difference between the UCP1-independent and the UCP1-dependent part of adrenergically induced O_2_ consumption (Fig. 7, *D*–*F*).

In Fig. 7*G*, we have compiled the responses of the UCP1-KO mice to the NE injection. As seen, the responses of the chow-, cafeteria-, and HFD-fed UCP1-KO mice fully overlap. Thus, in agreement with earlier conclusions (8), the UCP1-independent fraction of adrenergically induced thermogenesis is not recruitable by different diets. This means that there is no diet-recruitable UCP1-independent mechanism for adrenergically induced, diet-recruited thermogenesis either in BAT or elsewhere in the body.

By subtracting the average NE-induced response in UCP1-KO mice from the average response in WT mice, we calculated the UCP1-dependent thermogenesis, which was markedly increased in HFD- and cafeteria-fed mice compared with chow-fed mice (Fig. 7*H*). We thus conclude that both cafeteria and HFD feeding increases the thermogenic capacity of BAT, and this may imply that it is this augmented thermogenic capacity that protects WT mice from excessive obesity when they are exposed to obesity-inducing diets.

#### Cafeteria and HFD feeding also augment UCP1 amounts in 129SV/pas mice.

The most likely reason for the increased thermogenic response to NE injection observed above would be an increase in the total amount of UCP1 in the BAT. Therefore, at the end of the experiment, we examined the IBAT depot in the 129SV/pas mice.

**Fig. 8.**
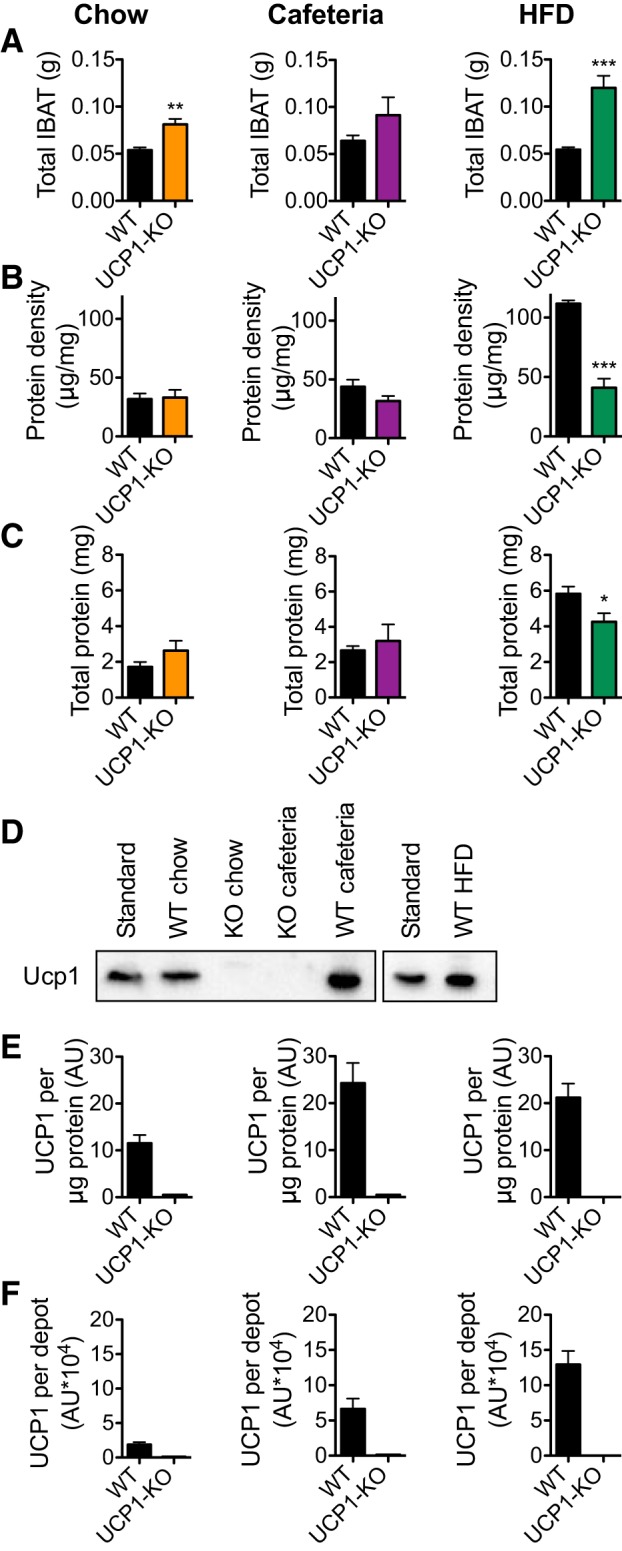
Total interscapular brown adipose tissue (IBAT) uncoupling protein (UCP1) protein levels are increased by cafeteria and high-fat diet (HFD) feeding. Data from the 129SV/pas mice, examined on experimental *day 31*. *A*: total IBAT tissue weights at the end of the experimental period of wild-type (WT) and UCP1-knockout (KO) mice fed a chow, cafeteria, or HFD. *B*: protein density of IBAT tissues as in *A*. *C*: total protein in IBAT tissues as in *A*. *D*: representative immunoblots showing UCP1 protein in IBAT homogenates. Standard = mixture of BAT homogenates. *E*: quantification of blots as in *D* showing IBAT UCP1/μg protein. *F*: calculation of total UCP1 protein/IBAT depot. Data are represented as means ± SE; *n* = 6. *P < 0.05, **P < 0.01, and ***P < 0.001; Student’s *t*-test.

Total IBAT tissue weight was generally increased in the UCP1-KO mice (Fig. 8*A*). The IBAT of WT HFD-fed mice had a significantly higher protein density than that of chow- and cafeteria-fed mice (*P* < 0.001), indicating that the tissue was less lipid filled in the presence of UCP1; no such effect was seen in the absence of UCP1 (Fig. 8*B*). The total protein content was higher both in the cafeteria-fed (*P* < 0.05) and in the HFD-fed (*P* < 0.001) WT mice than in the chow-fed mice, but almost no effect of diet was seen in the UCP1-KO mice (Fig. 8*C*).

Immunoblotting for UCP1 revealed a significant increase in UCP1 protein per microgram of BAT protein in cafeteria- and HFD-fed mice compared with chow-fed mice (*P* < 0.05) (Fig. 8, *D* and *E*). When the differences in total protein content of the tissues were taken into account, we found that the consumption of a cafeteria diet indeed increased total BAT UCP1 protein levels compared with chow-fed mice, and total BAT UCP1 protein levels were even higher in HFD-fed mice [WT chow vs. WT cafeteria (*P* < 0.05), WT cafeteria vs WT HFD (*P* < 0.05); Fig. 8*F*]. Thus, the increased thermogenic response to NE in the cafeteria- and HFD-fed mice versus the chow-fed mice is associated with a higher total content of UCP1 in the BAT.

#### BAT UCP1 protein amount correlates positively with fat mass and is associated with protection against further obesity.

**Fig. 9.**
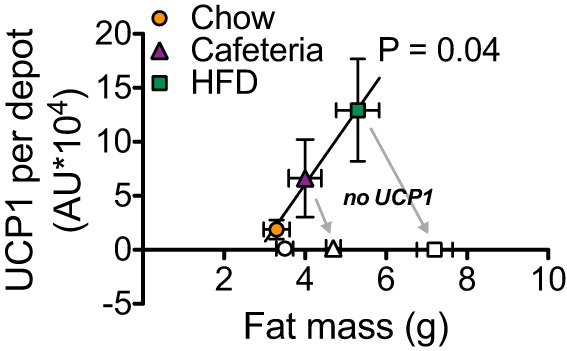
A high total interscapular brown adipose tissue (IBAT) uncoupling protein (UCP1) protein amount correlates with a high fat mass. Data from the 129SV/pas mice. Correlation in wild-type (WT) mice between total IBAT UCP1 protein levels as shown in Fig. 8*F* and fat mass as shown in Fig. 6 *D*–*F*. Gray arrows indicate the increase in fat mass due to the absence of UCP1. Data are represented as means ± SE; *n* = 6. Pearson’s correlation. AU, arbitrary units.

The total amount of UCP1 protein in the IBAT of cafeteria-fed mice was higher than that in chow-fed mice, and the amount in HFD-fed mice was even higher (Fig. 8*F*). At the same time, the total body fat mass was also higher in cafeteria-fed mice than in chow-fed mice and even higher in the HFD-fed mice (Fig. 6, *D*–*F*). Indeed, if the total amount of UCP1 is plotted as a function of total body fat mass, a clear positive correlation is found (Fig. 9), basically in extension of earlier observations for these diets on C57Bl/6 mice (7). This positive correlation may initially seem unexpected, given the generally accepted hypothesis that less BAT may augment the development of obesity. However, provided that this is a functional correlation, it indicates that UCP1 is recruited to an increasing extent as the mouse becomes more and more obese. The function of this would be that it would partly counteract further obesity. Indeed, as seen through the gray arrows in Fig. 9, in each diet state, the absence of UCP1 leads to an aggravated obesity, with this aggravation being larger the higher the amount of “missing” UCP1 is. Thus, even in an obesity-resistant mouse strain, obesity is induced and amplified in the absence of UCP1.

## DISCUSSION

In the present investigation, we demonstrate that the obesity-reducing effect of UCP1 is observable not only in an obesity-prone mouse strain but also in mouse strains that are recognized to be obesity resistant. Additionally, we find that metabolic effects of UCP1 may even be observable when no obesity is induced, in that case observable rather as the ability to retain body energy reserves based on a lowered energy intake.

In addition to generalizing the metabolic significance of UCP1 and BAT, this investigation also demonstrates that all diet-induced UCP1-dependent facultative thermogenesis originates from classical BAT (rather than brite/beige adipose tissue) and that no UCP1-independent mechanism for recruitable adrenergic thermogenesis exists, not even in obesity-resistant animals. That the metabolic effects of UCP1 are discernable even in these mice where the BAT is rather involuted (since the mice were kept at thermoneutrality) is of translational importance, as it implies that even minor amounts of UCP1 may affect metabolism and thus also obesity development in humans.

### 

#### UCP1 ablation induces obesity.

We show here that even in thermally humanized and innately obesity-resistant 129S mice, the consumption of Western-style diets induces diet-induced thermogenesis mediated by UCP1 protein in BAT; in the absence of this, obesity develops. The acquirement of this diet-induced thermogenesis is a successive process. In the WT mice, the higher food intake occurring when a cafeteria diet or a high-fat diet is offered (Figs. 2 and 6) leads to an increase in adiposity. An implication would be that this increase in adiposity (if the total fat mass exceeds 3 g; Fig. 9) is interpreted as an error signal and is in some way associated with an increase in the amount of UCP1. Indeed, UCP1 protein amount in IBAT and, in parallel, whole body, UCP1-dependent, NE-induced oxygen consumption is increased in response to HFD or cafeteria feeding (Figs. 5, 7, 8, 9). In contrast, the UCP1-independent component of NE-induced oxygen consumption does not vary depending on diet (Figs. 5, 7, and 8). The presence of the extra UCP1 results in a higher fraction of the ingested energy being combusted during each meal, and this reduces the amount of energy left for storage.

Correspondingly, the absence of UCP1 increases metabolic efficiency, which is visible as an increased fat accumulation on HFD or cafeteria diet feeding (Figs. 1, 2, and 6). The increased metabolic efficiency in 129S UCP1-KO mice is due to a decreased energy expenditure (Fig. 4). The development of obesity induced by the ablation of UCP1 progressively worsens, as the gap between the UCP1 amount that the animal should have, but lacks, widens (Fig. 9).

#### UCP1 and metabolic efficiency.

Whereas the data discussed above substantiate that UCP1 ablation causes increased obesity, the present investigation additionally demonstrates that even when obesity does not develop, such as when an obesity-resistant mouse is exposed only to a chow diet (Figs. 1 and 2), the mouse will protect its body weight by reducing the food intake. A similar tendency is seen with the mice exposed to the simplified cafeteria diet (Fig. 6*H*). This is a phenomenon observable only in obesity-resistant mice and indicates that these mice possess a very strong feedback from the body fat to suppress appetite. A further implication is that a fraction of the energy ingested is combusted through BAT activity, even under conditions when no physiological reason for BAT activity can be formulated (i.e., at thermoneutrality and in the absence of obesity).

#### Diet-induced adrenergic thermogenesis is mediated solely by UCP1 in BAT.

Although we demonstrate here that diet-induced thermogenesis is UCP1 dependent, it may not necessarily be localized to classical BAT. Indeed, certain white adipose tissue depots possess an ability to develop brown-like characteristics, including acquiring UCP1 gene expression (10, 17, 19, 30). These tissues are referred to as brite or beige adipose tissues, and the conversion is particularly observable in the inguinal adipose tissue depot. It has been hypothesized that this “browning” of certain white adipose tissue depots may contribute substantially to the UCP1-dependent lowering of metabolic efficiency (12, 15, 29). There would thus be a possibility that part of the UCP1-mediated diet-induced thermogenesis could take place in brite/beige adipose tissue. However, we demonstrate that UCP1 protein is not detectable in the ingWAT of 129S2/sv mice housed at thermoneutrality (Fig. 5) and is not induced by a high-fat diet. Similarly, the UCP1 levels in ingWAT of mice living at room temperature are actually reduced by a high-fat diet in both obesity-prone (AKR) and obesity-resistant (SWR) strains (20). Therefore, we conclude that UCP1-mediated, diet-induced thermogenesis occurs only in BAT.

In addition, there have been suggestions that there may be alternative adaptive thermogenic mechanisms apart from heat production through UCP1 (1, 16, 26). However, it is clear from our results that in UCP1-KO mice there is no effect of adaptation to the diets on NE-induced oxygen consumption (Fig. 7*G*). Thus, in 129S mice housed in the absence of thermal stress, diet-recruited, adrenergically induced thermogenesis is fully UCP1-dependent. Conversely, it may be discussed whether nonadrenergically induced, UCP1-independent means of diet-induced thermogenesis may exist. There is, e.g., a clear indication that the noninjected cafeteria-fed or high-fat diet-fed WT mice show a higher basal metabolic rate compared with chow-fed WT mice [in the anesthetized state; WT chow vs. WT cafeteria (*P* < 0.01), WT chow vs. WT HFD (*P* < 0.05); Fig. 7, *A*–*C*]. The reason for this is not known, but also, this type of thermogenic activity is not manifest in the absence of UCP1. This diet-induced thermogenesis is thus also UCP1 dependent.

Our data cannot fully exclude that minor UCP1-independent, nonadrenergic mechanisms for diet-induced thermogenesis may exist. Because even very small mismatches between energy intake and energy expenditure with time may result in measurable alterations in the amount of stored energy, such processes may be important, but none of the data obtained here in the obesity-resistant 129S mouse strain imply that it possesses any means of diet-induced thermogenesis that are UCP1 independent.

#### UCP1-mediated, diet-induced thermogenesis contributes to total energy expenditure.

Despite its minimal contribution to total body weight, adrenergically activated BAT may account for a significant part of total energy expenditure. Here, in 129S mice, BAT makes up only 0.25% of total body weight (assuming an average IBAT mass of 0.07 g and an average body weight of 28 g, as is generally observed in this study). Even if the additional BAT depots in the rodent are taken into account, BAT’s contribution to body weight would not exceed 1%. We show here that at thermoneutrality, the presence of this small tissue and its corresponding UCP1 is associated with an increase in energy expenditure of ∼15% upon HFD feeding (Fig. 4).

#### UCP1 versus obesity.

We find here and earlier that in mice there is a positive relationship between obesity and UCP1 and BAT amounts (7, 8), and there are similar indications in humans (23, 25). Whereas this initially may be considered paradoxical, given that UCP1 is supposed to counteract obesity, such a recruitment of the tissue is fully in agreement with a homeostatic role of the tissue. It would appear that the organism has a certain desired body fat mass content, and the further the body fat deviates from this, the larger the error signal that leads to more UCP1 becomes. That in its turn will increasingly counteract the development of further obesity. Thus, the recruitment of UCP1 is not a result of an analysis of the quality of the food as such but is only a homeostatic effector that identifies a deviation from the “set” body fat mass of 3 g. The further implication could be that the reported association between obesity and lack of UCP1 (14, 28) is an indication of a pathological state occurring when the recruitment of UCP1 is prevented due to altered hormone levels or misfunctions in the central regulation of BAT.

#### Human relevance.

Although understanding the phenomenon of diet-induced thermogenesis is a goal in itself, examination of this phenomenon has implications for human biology, since an augmentation of diet-induced thermogenesis in humans could counteract the development of obesity. At issue has been whether an inactivation of BAT could be causative for augmenting obesity even in humans or whether this effect would be restricted to a single metabolically atypical mouse strain: the C57Bl/6 strain. Indeed, had the obesity-inducing effect of UCP1 ablation been restricted to this obesity-prone mouse strain, the relevance of innate UCP1 for human obesity control would of course be nonexistent (although of course acquisition of active BAT in humans could still counteract the development of further obesity). By demonstrating the significance of UCP1 in a mouse strain that is so markedly metabolically opposite of the obesity-prone C57Bl/6 mouse strain, we infer here that BAT and UCP1 are of metabolic significance principally in all mouse strains.

However, it has been implied that humans possess so little and so involuted BAT that the metabolic role would be negligible. Nonetheless, the relative amount of BAT in humans is of the same order of magnitude as that found here in mice (∼250 g in a 70-kg human: ∼0.4%). Furthermore, the BAT of humans, although it appears atrophied, in reality phenocopies the classical BAT of mature mice living at thermoneutrality and eating a Western style diet (de Jong JM, Sun W, Pires N, Frontini A, Balaz M, Petrovic K, Fischer AW, Bokhari M, Niemi T, Nuutila P, Cinti S, Virtanen K, Cannon B, Nedergaard J, Wolfrum C, and Petrovic N, unpublished observations). Because this small relative amount of BAT, with its atrophied appearance, is still a significant factor for the obesity resistance of this mouse strain, this implies that also relatively small amounts of relatively unrecruited BAT could be of metabolic significance in humans and that the maintenance or even recruitment of this tissue may be of benefit to human health.

## GRANTS

This project was supported by grants from the Swedish Research Council to B. Cannon and J. Nedergaard.

## DISCLOSURES

No conflicts of interest, financial or otherwise, are declared by the authors.

## AUTHOR CONTRIBUTIONS

I.H.L., H.M.F., G.v.E., B.C., and J.N. conceived and designed research; I.H.L. and H.M.F. performed experiments; I.H.L. and H.M.F. analyzed data; I.H.L., H.M.F., G.v.E., B.C., and J.N. interpreted results of experiments; I.H.L. prepared figures; I.H.L. drafted manuscript; I.H.L., B.C., and J.N. edited and revised manuscript; I.H.L., H.M.F., G.v.E., B.C., and J.N. approved final version of manuscript.
